# Functional lower extremity strength influences stepping strategy in community-dwelling older adults during single and dual-task walking

**DOI:** 10.1038/s41598-024-64293-0

**Published:** 2024-06-11

**Authors:** Brandon M. Peoples, Kenneth D. Harrison, Keven G. Santamaria-Guzman, Silvia E. Campos-Vargas, Patrick G. Monaghan, Jaimie A. Roper

**Affiliations:** 1 School of Kinesiology, Auburn University, Auburn, AL USA; 2 College of Pharmacy and Health Sciences, Wayne State University, Detroit, MI USA; 3 University of Costa Rica, San Pedro, San Jose Province Costa Rica

**Keywords:** Mobility, Lower extremity strength, Gait, Dual-task, Stepping strategy, Cognitive ageing, Ageing

## Abstract

As age increases, a decline in lower extremity strength leads to reduced mobility and increased fall risks. This decline outpaces the age-related reduction in muscle mass, resulting in mobility limitations. Older adults with varying degrees of mobility-disability use different stepping strategies. However, the link between functional lower extremity strength and stepping strategy is unknown. Therefore, understanding how age-related reductions in functional lower extremity strength influence stepping strategy is vital to unraveling mobility limitations. Twenty participants (17F, 72 ± 6 years) were recruited and tested at a local community event. Participants were outfitted with inertial measurement units (IMU) and walked across a pressurized walkway under single and dual motor task conditions (walking with and without carrying a tray with water) at their usual and fast speeds. Participants were dichotomized into normal (11) or low functional strength groups (9) based on age-specific normative cutoffs using the instrumented 5-repetition Sit-to-Stand test duration. Our study reveals that older adults with normal strength prefer adjusting their step time during walking tasks, while those with reduced strength do not exhibit a preferred stepping strategy. This study provides valuable insights into the influence of functional lower extremity strength on stepping strategy in community-dwelling older adults during simple and complex walking tasks. These findings could aid in diagnosing gait deviations and developing appropriate treatment or management plans for mobility disability in older adults.

## Introduction

Maintaining mobility is vital for older adults to preserve their independence, self-reliance, community involvement, and overall health. Nevertheless, the aging process has a notable impact on this ability. As individuals cross their fifties, they may experience a gradual decline in their lower extremity strength, leading to reduced functional mobility, slower walking speeds, increased sedentary behavior, social isolation, higher fall risks, and a decline in their quality of life across physical, cognitive, emotional, and social domains^1–3^. Alarmingly, lower extremity strength deteriorates faster than age-related reductions in muscle mass, prompting mobility limitations since walking requires supporting body weight through sit-to-stand transitions and propelling the body forward during walking using the lower body muscles^3–5^. Thus, addressing deficits in lower extremity strength as the population ages is crucial to understanding the complex relationship between decreased functional lower extremity strength, gait, and mobility-disability.

Functional lower extremity strength plays a critical role in preserving mobility but inevitably declines with advanced age, resulting in a nearly three-fold increase in fall risks in older adults and costing over $50 billion in annual geriatric injury expenditures from government programs such as Medicare^6,7^. Quantifying decrements to functional lower extremity strength before outright mobility-disability occurs is critical but requires direct assessments linking functional lower extremity strength to gait safety. The 5-repetition Sit-to-Stand test (5xSTS) fills this need since it is a clinically feasible test of functional lower extremity strength that holds validity and strongly predicts mobility limitations in older adults^8–11^. Poor performance on the 5xSTS is associated with reduced gait speed, functional impairment, frailty, and fall risk in community-dwelling adults^1,8, 11–14^.

Stepping strategy is essential when examining older adults’ normal and dual-task walking patterns. There are three main stepping strategies: neutral, step-time dominant, and step-length dominant. Neutral stepping strategies involve a balanced contribution of step time and step length adjustments to regulate walking pace^15^. Step-time dominant strategies prioritize the modulation of step duration to increase or decrease walking speed. In contrast, step-length dominant strategies focus on adjusting step length to navigate the environment and change pace^15^. Older adults at risk for mobility disability with reduced functional lower extremity strength, measured by the 5xSTS, rely more on a step-time dominant strategy to adjust walking speed without the presence of a secondary task^15^. To understand how older adults navigate their environment comprehensively, we need to consider not only their stepping strategies and functional lower extremity strength but also their ability to perform multiple tasks simultaneously while walking^16,17^. Therefore, examining the relationship between functional lower extremity strength, stepping strategy, and task complexity in older adults during walking provides insights into how age affects this complex interaction.

This study aims to investigate whether functional lower extremity strength affects stepping strategy in community-dwelling older adults across normal and dual-task walking. We hypothesize: (1) Older adults with reduced functional lower extremity strength will use a step-time dominant stepping strategy compared to those with normal strength, and (2) each group will have a preferred stepping strategy during walking conditions where multitasking is required. Thus, investigating functional lower extremity strength and stepping strategy can enhance our understanding of the factors that impact gait stability and help devise effective interventions to address mobility disability proactively.

## Methods

### Participants

Our study’s participant recruitment and data collection were conducted at a local community health fair event instead of a laboratory. This was done to improve ecological validity and demonstrate the feasibility of implementing assessment procedures in a practical, real-world context. The recruitment and data collection took place over two consecutive years (2022 and 2023) as part of our ongoing research engagement and service partnership to address health needs in a rural region. Our study focused on a sample from an underserved rural community. It was in response to a call from the National Institute of Aging (NIA) workshop on age-related changes in gait biomechanics. The workshop stressed the importance of diverse sampling and building community partnerships^18^. Twenty older adults (17F, 72 ± 6) living in the Lee County community participated in this study during a local health fair. Participants who were free from lower extremity injuries such as bone fractures, muscle strains, and joint dislocations were included in the study. Participants who reported any neurological disease such as Parkinson’s disease, Essential Tremor, Multiple Sclerosis, Stroke, or Traumatic Brain injury were excluded from the study. This study did not include participants who used a walking aid such as a cane or walker. Two participants reported using five or more different medications regularly, but their medication and disease status were not tracked. Information on the total number of participants recruited each year can be found in Table 1 below.

**Table 1:** Total participant recruited each year by race.

Year	Race	*n*	*%*
2022	Asian	1	9.1
	Black or African American	3	27.3
	White or Caucasian	7	63.6
	Missing	0	0
	Total	11	100
2023	Asian	0	0
	Black or African American	5	55.6
	White or Caucasian	4	44.4
	Missing	0	0
	Total	9	100

### Material

The Auburn University Institutional Review Board approved this study on the basis of minimal risk, and a waiver for informed consent was granted. Before participating in this study, all participants were given an IRB-approved information letter with a detailed explanation of the study’s purpose, procedures, potential risks, and benefits. After reviewing the information letter, participants underwent a series of assessments, including demographic questionnaires, a functional lower extremity strength assessment, and walking assessments. The questionnaire included questions about their demographic information, living status, education, sleep habits, retrospective fall history, and self-reported physical activity history. Fear of falling was measured using the Fall Efficacy Scale (FES) developed by Tinetti et al. (1990), and barriers to physical activity were assessed using the CDC Barrier to Being Active Quiz (BBAQ) (Control & Prevention, 2013)^19^. The study was conducted according to the ethical principles of the Declaration of Helsinki.

### Functional Lower Extremity Strength measured by the Instrumented 5-Rep Sit-to-Stand (i5xSTS)

The i5xSTS device was employed to measure the functional strength of the lower extremities using wireless inertial measurement units (IMU) from APDM Opals (APDM Inc, Portland, OR). The Opal sensor has triaxial accelerometers, gyroscopes, and magnetometers, capturing signal data at 128 Hz. To obtain readings, researchers placed six IMUs at four different body locations: (1) atop the sternum centered over the manubrium, (2) on the superior aspect of the posterior sacral surface (below the fifth lumbar vertebrae L5), (3) on the posterior aspect of the distal radius and ulna, and (4) on the dorsal surface of the foot, centered on the intermediate and lateral cuneiform. Participants were instructed to sit with their backs against the chair, arms crossed, and hands touching the anterior aspect of their deltoid. They were then asked to stand and sit as quickly as possible for five repetitions. The duration of this exercise was used to determine the strength status of the participants in this study.

### Gait testing

All participants completed the walking trials on level ground at a local sportsplex basketball court adjacent to the main health fair venues and were exposed to approximately 75 dB of sound. We did not control footwear; however, participants were not permitted to complete testing barefooted. Each year, all walking assessments were done in the morning during vendor exhibitions, demonstrations, and community events to reflect real-world complexity. Before walking, participants were seated for at least ten minutes to complete the questionnaires. Participants were then asked to walk under four conditions during the community health fair. These were: (1) Walking at their normal speed, (2) Walking at their fastest speed, (3) Motoric Dual Task at their normal speed (Walking at their normal speed while holding a tray with a cup of water), and (4) Motoric Dual Task at their fastest feed (Walking at their fastest speed while holding a tray with a cup of water. Researchers instructed the participants to walk at a “comfortable, natural walking speed” or the “fastest, safe walking speed without jogging or running,” as prompted. Additionally, the researchers provided non-prioritizing instructions while the participants walked with the tray to prevent participants from prioritizing the tray task over the walking task and vice versa. For analysis, the average of all steps was calculated based on a single trial completed under each condition. The GAITRite instrumented walkway was used to capture all spatial and temporal gait cycle parameters used for this study. Our GAITRite system (GAITRite Gold, CIR Systems, Clifton, NJ) consists of an electronic walkway approximately 8.2 m long, connected to a personal computer via an interface cable. The walkway comprises a series of sensor pads inserted in a grid formation between a layer of vinyl (top cover) and foam rubber (bottom cover). The active area of the walkway is 61 cm wide and 732 cm long, while the sensors are placed 1.27 cm apart, consisting of a total of 27,648 sensors that are activated by mechanical pressure. The data from the activated sensors is collected by a series of onboard processors and transferred to the computer through a serial port. The system has a sampling rate of 80 Hz.

## Data analysis

### Functional Lower Extremity Strength measured by the Instrumented 5-Rep Sit-to-Stand (i5xSTS)

i5xSTS was collected from APDM Opals, and participant data was processed using Moveo Explorer version 1.0.0.202206 (APDM Inc, Portland, OR). Total duration was calculated using the average duration of the sit-to-stand and stand-to-sit transitions. Participants were classified as low strength (LS) if their 5xSTS duration exceeded normative performance values for their age range of 11.4 s (60–69 years), 12.6 s (70–79 years), and 14.8 s (80–89 years)^20^.

### Gait cycle parameters

Gait speed, step length, and step time for all walking conditions were exported from the GAITRite software (GAITRite, CIR Systems Inc., Clifton, NJ). Step length was measured using the heel center of the current footprint to the heel center of the previous footprint on the opposite foot. Step time is the elapsed time from the first contact of one foot to the first contact of the opposite foot. Gait Speed was measured using the distance traveled divided by the total ambulation time. Step length and step time from each condition was used to calculate the stepping strategy measured by Length–Time Difference (see below).

### Length–Time Difference (LTD)

LTD was derived from a previous study evaluating how strategy, adjustments to cadence or stride length when walking at a normal speed and fast speed, influence lower extremity joint moments^21^. The equation used to calculate strategy for cadence and stride length are below:

Ardestani’s Strategy equation (1)

$$\Delta Cadence_{i} = \left( {\frac{{Cadence_{Fast} - Cadence_{Normal} }}{{Cadence_{Normal} }} } \right)*100\% i{ } = {\text{ number\;of\;subjects}}$$

$$\Delta Stride_{i} = \left( {\frac{{Stride_{Fast} - Stride_{Normal} }}{{Stride_{Normal} }} } \right)*100\%$$

**Figure 1:**
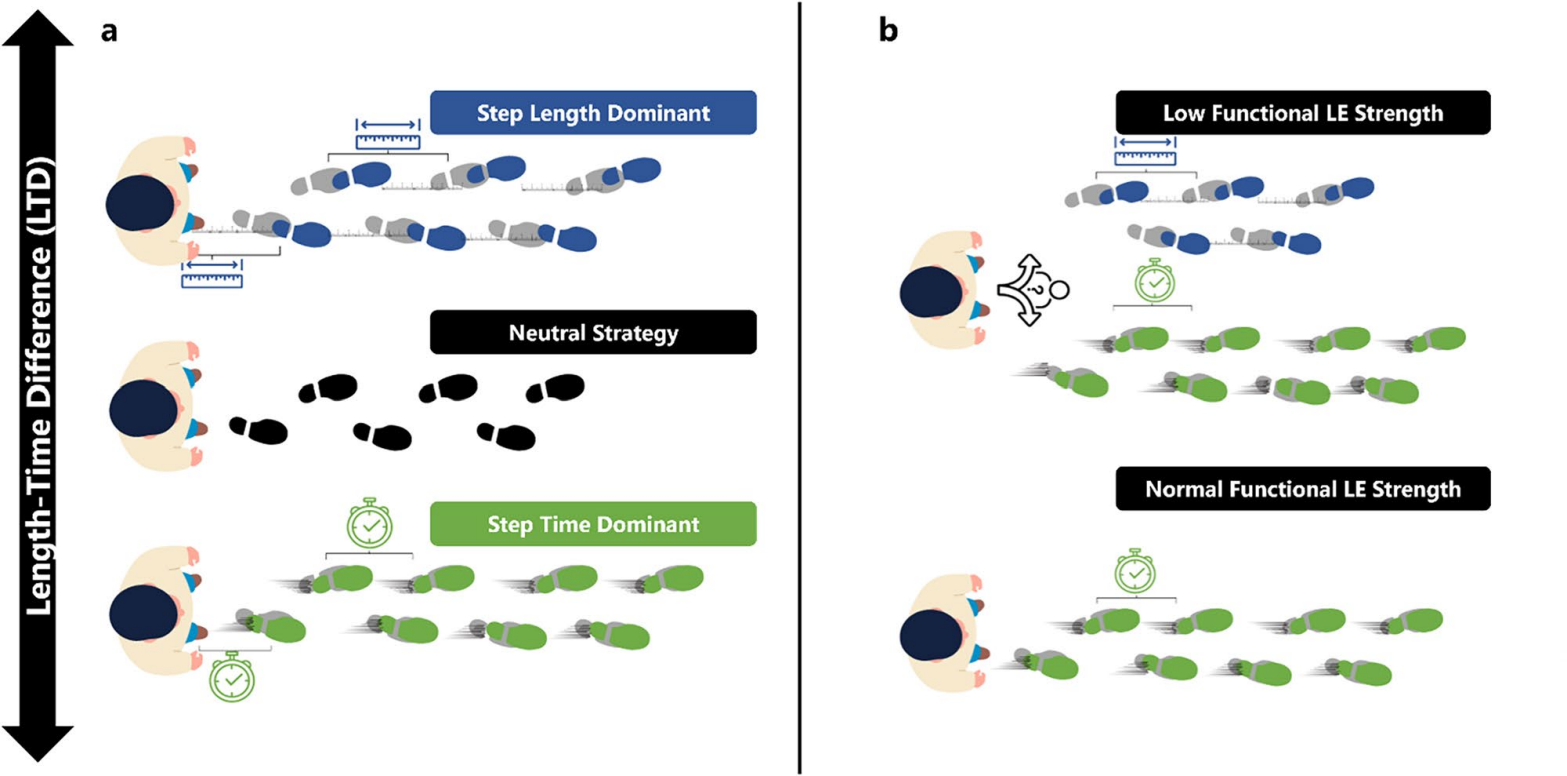
Schematics demonstrating the different stepping strategies used when comparing multiple walking conditions. (**a**) Schematic demonstrating the different stepping strategies used when comparing multiple walking conditions (i.e., walking at a fast speed vs walking at a normal speed), (**b**) Depicts the strategy chosen by older adults with normal functional lower extremity strength measured through the 5-repetition sit-to-stand compared to older adults with low functional lower extremity strength. Older adults with normal functional lower extremity strength preferred adjusting step time across single and dual-task walking tasks compared to older adults with reduced functional lower extremity strength who did not have a preferred strategy.

LTD represents relative change in step length and step time when comparing two conditions (i.e. fast vs preferred. An LTD > 0 indicates a step length dominant strategy (increasing step length), an LTD < 0 indicates a step time dominant strategy, while an LTD = 0 indicates a neutral strategy, implying equal contribution from both step time and step length (see Fig. 1)^21,15^.

Length–Time Difference equation (2)

$$LTD = \left( {\frac{{Length_{Fast} - Length_{preferred} }}{{length_{preferred} }} } \right) + \left( {\frac{{Time_{Fast} - Time_{preferred} }}{{Time_{preferred} }}} \right)*100\%$$

Modified Length–Time Difference equation (3)

$$LTD = \left( {\frac{{Length_{Tray + Walk Speed} - Length_{Walk Speed} }}{{length_{Walk Speed} }} } \right) + \left( {\frac{{Time_{Tray + Walk Speed} - Time_{Walk Speed} }}{{Time_{Walk Speed} }}} \right)*100\%$$

Following the equations above, we calculated the stepping strategy for four distinct comparisons: Walking at a Fast Speed vs Walking at a Normal Speed (Walking Comparison 1), Motoric Dual Task at a Fast Walking Speed vs Motoric Dual Task at a Normal Walking Speed (Walking Comparison 2), Motoric Dual Task at a Normal Walking Speed vs Walking at a Normal Speed (Walking Comparison 3), and Motoric Dual Task at a Fast Walking Speed vs Walking at a Fast Speed (Walking Comparison 4).

### Statistical analysis

#### Power analysis

An a priori power analysis was conducted using G*Power version 3.1.9.7^22^ to attain 80% power and determine the minimum sample size based on data from^15^, which observed nearly large effect size (*d* = 0.77) between the 5-repetition chair stand duration of the Short Physical Performance Battery and stepping strategy using Length–Time difference in older adults with mobility limitations. With the significance criterion set at *α* = 0.05 and power = 0.80, the minimum sample size needed with this effect size is *N* = 12 for repeated measures ANOVA.

### Chi square test for independence

A chi-square test of independence was conducted to compare education levels and sex between groups.

### Independent samples T-test

An independent samples t-test was conducted to compare age, daily physical activity minutes, education level, height, mass, 5 × sit-to-stand test duration, overall gait speed, sit-to-stand duration, stand-to-sit duration, sit-to-stand angle, stand-to-sit angle, step count, step length, step time, and Falls Efficacy Scale (FES) score between the low strength and normal strength groups. The standardized mean difference between the group means was reported using Hedges’ g due to the small sample size since it has a correction for a smaller sample size. Hedges’ g represents the extent of difference between the groups regarding standard deviations^23^. Levene’s test for equality of variances was conducted before running the independent t-test on all group comparators. The results of Levene's test were not statistically significant (p > 0.05), indicating that the assumption of homogeneity of variances was met for all variables.

### 2 × 4 repeated measures ANOVA

A 2 × 4 repeated measures ANOVA was conducted to examine the effect of group (Low vs. Normal) and walking comparisons: Walking at a Fast Speed vs Walking at a Normal Speed (Walking Comparison 1), Motoric Dual Task at a Fast Walking Speed vs Motoric Dual Task at a Normal Walking Speed (Walking Comparison 2), Motoric Dual Task at a Normal Walking Speed vs Walking at a Normal Speed (Walking Comparison 3), and Motoric Dual Task at a Fast Walking Speed vs Walking at a Fast Speed (Walking Comparison 4) measured by LTD. Mauchly’s test indicated that the assumption of sphericity was violated for condition (p < 0.05); therefore, we used Greenhouse–Geisser correction. Post-hoc test comparisons were performed using the Tukey HSD test. All analyses were performed in JASP (Version 0.18.3; JASP Team, 2024) which uses R computational engine.

## Results

### Independent samples T-tests

**Figure 2:**
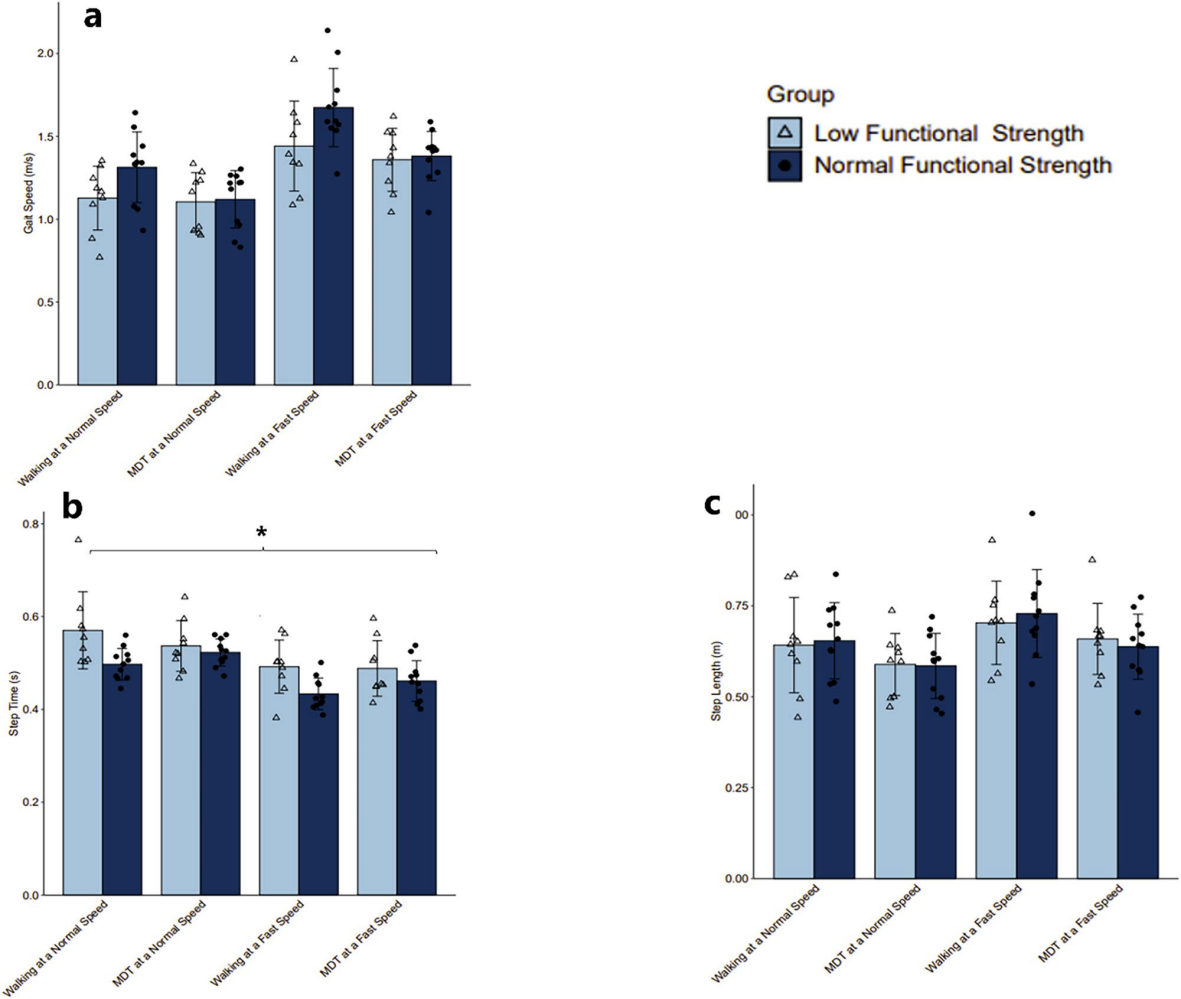
(**a**) Bar graph of mean gait speed (m/s) across conditions for each group with SE, (**b**) bar graph of mean step time (s) across conditions for each group with SE, * indicates p < 0.05, (**c**) bar graph of mean step length (m) across conditions for each group with SE.

An independent samples t-test was conducted to compare differences in group characteristics summarized in Table 2. There was no significant difference in age, sex, education level, daily physical activity, and Falls Efficacy Scale (FES). Further, there was no significant difference in gait speed, step length, step count, or stand-to-sit transition measures. However, significant differences were observed in several key measures. Most notably, the low-strength group demonstrated poorer performance on the instrumented 5xSit-to-Stand (5xSTS) test, which may be attributed to longer sit-stand-stand transition durations and greater Sit-to-Stand lean angles. Interestingly, the low-strength group exhibited a significantly longer step time across all walking conditions illustrated in Fig. 2.

**Table 2:** Descriptive statistics and independent samples t-test results comparing group characteristics.

	Low strength		Normal strength		*p*	Hedges’ g
Age (years)	74 ± 7	65–87	69 ± 5	61–78	0.07	0.84
Education level	9		11		0.76*	
HS Diploma or GED	2		3			
Associate’s or Trade Degree	4		3			
Bachelor’s Degree	2		2			
Master’s Degree	1		3			
Sex (M/F)	2M/7F		1M/10F		0.41*	
Daily PA Minutes	41 ± 20	20–60	49 ± 21	20–90	0.51	− 0.35
FES Score	17 ± 8	10–29	14 ± 6	10–30	0.36	0.46
Gait Speed (m/s)	1.26 ± 0.3	0.77–1.96	1.37 ± 0.3	0.83–2.1	0.06	− 0.43
Height (m)	1.64 ± 0.1	1.45–1.7	1.61 ± 0.1	1.47–1.8	0.58	0.27
Mass (kg)	84.1 ± 15.5	63.4–113.8	74.7 ± 14.6	52.6–101.3	0.24	0.62
5xSTS duration (s)	19.3 ± 4.7	15.3–27.6	11.3 ± 2.2	7.7–14.1	** < 0.001**	2.27
Sit-to stand duration (s)	1.4 ± 0.6	0.9–2.8	0.9 ± 0.17	0.7–1.2	**0.01**	1.39
Stand-to-sit duration (s)	0.90 ± 0.4	0.6–1.8	0.75 ± 0.3	0.6–1.5	0.32	0.46
Sit-to-stand lean angle (degrees)	57 ± 28	21–104	29 ± 6	21–38	**0.01**	1.41
Stand-to-sit lean angle (degrees)	32 ± 10	14–101	28 ± 6	23–28	0.08	0.83
Step count	6 ± 1	3–9	6 ± 1	3–8	0.93	− 0.02
Step length (m)	0.65 ± 0.11	0.44–0.93	0.65 ± 0.11	0.45–1.0	0.90	− 0.27
Step time (s)	0.52 ± 0.07	0.38–0.77	0.48 ± 0.05	0.39–0.56	**0.002**	0.72

*p-*values in bold indicate statistically significant differences between groups. Values indicate mean ± sd. Values indicate range: minimum–maximum.

M, male; F, female. FES, Falls Efficacy Scale; PA, physical activity.

*Indicates Chi-Square test of independence results.

### 2 × 4 repeated measures ANOVA

**Figure 3:**
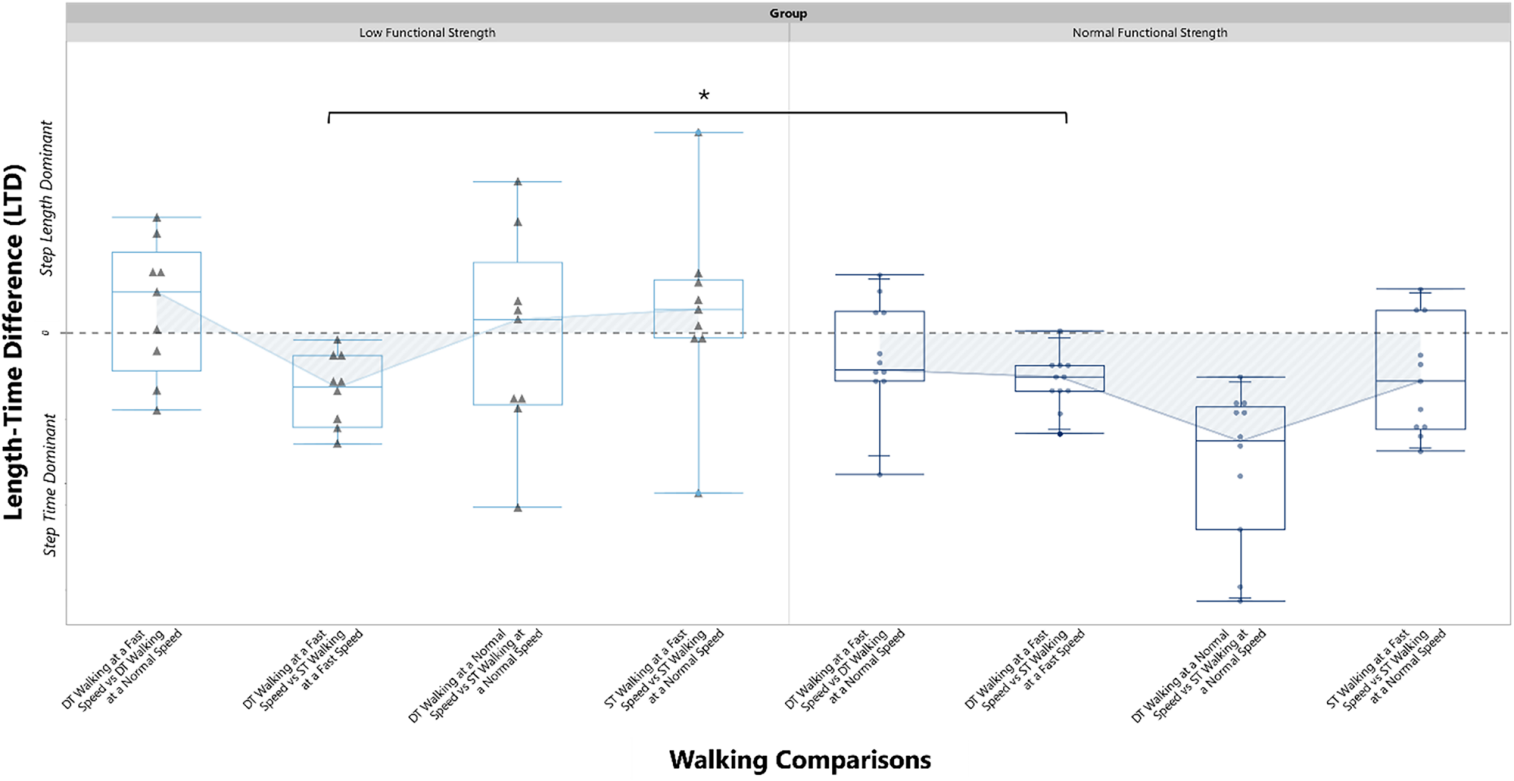
Box plots of stepping strategy across walking comparison for older adults with low functional lower extremity strength and normal functional lower extremity strength.

A 2 × 4 repeated measures ANOVA showed statistically significant differences in the main effects of stepping strategy, functional lower extremity strength (Low vs. Normal) and interaction between functional lower extremity strength and stepping strategy measured by LTD, summarized in Table 3. The normal strength group showed a clear preference for a step time dominant strategy compared to the low strength group. The low strength group vacillated between step length and step time across walking comparisons illustrated in Fig. 3.

**Table 3:** Main effects of repeated measures ANOVA.

Factor	*F* (df)	*p*	*η*^2^_*p*_
Walking comparison	5.21 (2,36)	0.01	0.22
Strength status	11.77 (1,18)	0.003	0.40
Walking comparison * Strength status	3.49 (2,36)	0.04	0.16

Post hoc analysis with a Tukey’s HSD revealed there was a mean difference between the Normal Strength Walking and Low Strength groups during Walking Comparison 3 (p < 0.001). Tests of simple main effects showed the interaction was driven by differences between strength groups in Walking Comparison 3 (Motoric Dual Task at a Normal Walking Speed vs Walking at a Normal Speed), summarized in Table 4.

**Table 4:** Simple main effects—strength status.

Level of condition	Sum of squares	df	Mean square	F	p
Walking comparison 1	310.211	1	310.211	4.057	0.059
Walking comparison 2	147.660	1	147.660	2.933	0.104
Walking comparison 3	1025.323	1	1025.323	9.661	**0.006**
Walking comparison 4	4.166	1	4.166	0.308	0.586

Significant values are in [bold].

## Discussion

The present study investigated whether functional lower extremity strength influences stepping strategy in community-dwelling older adults during simple and complex walking. Firstly, our main findings are that older adults with normal lower extremity strength preferred adjusting their step time when walking in single-task and dual-task conditions. Secondly, older adults with reduced functional strength did not exhibit a preferred stepping strategy, either with or without a motoric dual task (i.e., walking while holding a tray and balancing a cup of water). Lastly, the motoric dual task mostly influenced the interaction of the stepping strategy.

Our findings demonstrate that adults with reduced functional lower strength lack a preferred stepping strategy across all walking comparisons, whereas stronger older adults prioritize adjusting step timing over step length. Adjusting step timing may be a safer compensation for older adults to regulate walking given age-related declines in neuromuscular control, balance, proprioception, and reaction time^24,25^. Relying on adjusting step time likely reduces instability by bringing the center of mass closer to the leading foot to improve stability^26^. This process is regulated by the integration of interoceptive (vestibular system) and exteroceptive (visual and somatosensory systems) sensory feedback^27,28^. The vestibular system detects head position and orientation, while the visual and somatosensory systems provide information about the environment and the body’s segmental positions concerning the environment^29^. As people age, they often experience a decline in proprioception, an important part of the somatosensory system^30^. Decreased proprioception may lead to increased reliance on vestibular and visual feedback. The central nervous system processes this sensory information to generate the appropriate motor response for making adaptive changes in walking, such as adjusting step time. In older adults with normal functional lower extremity strength, adjusting step timing may enable smaller, more subtle changes by reducing the energy cost associated with altering step length through muscle activation and processing proprioceptive information on joint angles, allowing more gradual adaptations when presented with a second task. On the other hand, older adults with reduced functional lower extremity strength may have to alter step length to maintain stability when presented with a second task.

Contrary to our hypothesis, the low strength group did not demonstrate a step time strategy. Upon examination of the individual data, over half of the people in the low strength group utilized a step length strategy. Our findings disagree with Baudendistel et al.^15^ who reported in a sample of people with mobility disability, a longer sit to stand time relates to more step timing adjustments when comparing a walking trial at a comfortable pace versus a walking trial at a faster pace. Our differences between studies can be explained by the walking conditions used, and population sampled. Specifically, Baudendistel et al., recruited people who met requirements for mobility disability, as determined by physical inactivity, worse physical function, and slower preferred gait speed. Our study did not have the same exclusion and inclusion criteria, and we recruited people at a local health fair hosted in a community recreation center, and 13 of 20 participants reported being physically active^31^. Additionally, Baudendistel et al., compared a single task preferred-walking speed to a single task fast-walking speed. The interaction effect in our study reveals that functional lower extremity strength impacts stepping strategy primarily when attention is divided during walking—an essential skill for navigation and avoidance of fall risk hazards^32^. Interestingly, older adults in the low functional strength group would be considered at increased fall risk based on their instrumented 5 × Sit-to-Stand performance. This provides insights into the stepping strategies employed by older adults at heightened risk of falling. Our findings are consistent with the systematic review by Sherrington and colleagues, who found high-certainty evidence that exercise programs focusing on balance and functional training reduce the rate of falls by 24% in older adults living in the community^33^. Moreover, their review suggests that the most effective fall prevention programs combine balance and functional exercises with resistance training, leading to a 34% reduction in falls. This underscores the importance of targeting both lower extremity strength and functional performance to optimize stepping strategies and mitigate fall risk in older adults.

The relationship between functional lower extremity strength, stepping strategy, and attentional resources has important implications for fall prevention in older adults. Akin and colleagues found that both motor-cognitive and motor-motor dual-task training improved balance in older adults, although only motor-cognitive training enhanced walking functionality following 8-weeks of dual-task training^34^. Norouzi and colleagues further demonstrated that motor-cognitive dual-task training combining resistance exercises with cognitive tasks resulted in greater improvements in balance and working memory compared to motor-motor dual-task training (resistance training plus motor skill training) in older men^35^. Importantly, these cognitive and motor benefits were maintained 12 weeks after the intervention. Moreover, Sherrington and colleagues systematic review found high-certainty evidence that multicomponent exercise programs, particularly those including balance, functional, and resistance training, can reduce falls by 23–34% in community-dwelling older adults^33^. By incorporating functional strength training and dual-task practice into fall prevention programs, clinicians can effectively target the key physiological and cognitive factors contributing to gait instability and fall risk in older adults. This approach is particularly advantageous as it requires minimal equipment and can be easily implemented in various settings, including community centers, retirement homes, and rehabilitation clinics. Moreover, the low-cost nature of this intervention makes it accessible to a wide range of older adults, regardless of their socioeconomic status. Future research should explore how this multimodal approach impacts gait variability and dual-task performance in this population, as well as its long-term effectiveness in reducing fall-related injuries and healthcare costs.

A strength of our study is its community-based approach, which fosters inclusivity compared to traditional university settings. By engaging individuals in community settings, we enhance the generalizability of our findings and address the limitations associated with convenience samples from local university communities. Actively seeking participation from the community ensures our research reflects a broader spectrum of experiences and enriches our results’ validity and applicability^18^. This collaborative approach, rooted in the principles of community engagement, fosters collaboration, enhances diversity, and ensures interventions meet specific needs. Firstly, we suggest developing standardized protocols and best practices for engaging diverse older adult populations in community settings to ensure consistent and effective recruitment strategies. Secondly, we suggest researchers should collaborate to conduct larger-scale community-based studies involving single and dual-task walking assessments across multiple sites to enhance the generalizability and validity of findings further. Thirdly, we suggest researchers explore the potential of community-based participatory research approaches to actively involve older adults in designing, implementing, and evaluating fall prevention programs, fostering a sense of empowerment and ownership. Finally, future research should explore how this multimodal approach impacts gait variability and dual-task performance in this population, as well as its long-term effectiveness in reducing fall-related injuries and healthcare costs.

There are a few limitations that should be considered when interpreting the findings of our study. Firstly, most participants were female, which could affect the generalizability of the results as men may have different stepping strategies based on their functional lower extremity strength. Despite our efforts to recruit more male participants, we received fewer male volunteers each year. Anecdotally, we noticed that older men were more likely to participate in the study if their spouses were also involved. Secondly, while participants were given enough rest before their lower extremity strength and walking assessments, we did not control for any physical activity they may have engaged in on the day of the assessment. This could have affected the results. Thirdly, we only asked participants about their regular use of five or more medications, but we did not track the specific medications that could have affected their gait performance. Two participants in the normal strength group reported taking five or more medications, but information on non-neurological medical conditions was not obtained. Lastly, we did not measure executive function directly within each group, which could have provided further insights into the differences observed between groups during the dual-task conditions.

## Conclusion

The present study investigated whether functional lower extremity strength influences stepping strategy in community-dwelling older adults during simple and complex walking. Our study found that functional lower extremity strength, as assessed by the 5-repetition Sit-to-Stand instrument, influences stepping strategy in community-dwelling older adults. Older adults with normal functional lower extremity strength utilize a step-time dominant strategy, adjusting step time under normal and dual-task conditions. In contrast, older adults with reduced lower extremity strength lack a clear preference for modulating step length or time to walk faster. Future research should investigate if lower extremity-focused functional strength training programs can yield a preferred stepping strategy in older adults. This could provide more insight into the importance of maintaining functional lower extremity strength to improve mobility and enhance quality of life.

## Data Availability

The datasets generated during and/or analyzed during the current study are available from the corresponding author upon reasonable request.
